# Associations between race, *APOE* genotype, cognition, and mortality among urban middle-aged white and African American adults

**DOI:** 10.1038/s41598-021-98117-2

**Published:** 2021-10-06

**Authors:** Jordan Weiss, Sharmin Hossain, Ana I. Maldonado, Botong Shen, Hind A. Beydoun, Mika Kivimaki, Michele K. Evans, Alan B. Zonderman, May A. Beydoun

**Affiliations:** 1 Department of Demography, University of California, Berkeley, Berkeley, CA USA; 2 Laboratory of Epidemiology and Population Sciences, NIA/NIH/IRP, Baltimore, MD USA; 3 Department of Psychology, University of Maryland Baltimore County, Catonsville, MD USA; 4 Department of Research Programs, Fort Belvoir Community Hospital, Fort Belvoir, VA USA; 5 Department of Epidemiology and Public Health, University College London, London, UK; 6 NIH Biomedical Research Center, National Institute on Aging, IRP, 251 Bayview Blvd., Suite 100, Room #: 04B118, Baltimore, MD 21224 USA

**Keywords:** Genetics, Psychology, Biomarkers, Cardiology, Neurology, Risk factors

## Abstract

We examined associations between cognition and mortality and how these relationships vary by race and Apolipoprotein E (*APOE*) genotype, in a longitudinal study of 2346 middle-aged White and African American adults (30–64 years at baseline) from the Healthy Aging in Neighborhoods of Diversity across the Life Span cohort study. Baseline cognition spanned global mental status, and several domains obtained using principal components analysis (PCA; PCA1: verbal memory/fluency; PCA2: attention/working memory; PCA3: executive function/visuo-spatial abilities). Cox regression models evaluated associations between cognition and all-cause and cardiovascular disease (CVD)-mortality. Interactions between cognition and *APOE2* as well as *APOE4* allelic dose were tested, and race was a key effect modifier. Higher *APOE4* dose was associated with increased CVD-mortality (hazard ratio [HR] per allele = 1.37; 95% CI 1.01–1.86, p = 0.041); *APOE2* dosage’s association with CVD-mortality was non-significant (HR = 0.60; 95% CI 0.35–1.03, p = 0.065). Higher PCA3 was associated with lower all-cause (HR = 0.93; 95% CI 0.87–0.99, p = 0.030) and CVD (HR = 0.85; 95% CI 0.77–0.95, p = 0.001) mortality risks, the latter association being more pronounced among Whites. PCA2 interacted synergistically with *APOE2* dosage, reducing risks for all-cause mortality (PCA2 × *APOE2*: − 0.33 ± 0.13, *p* = 0.010) and CVD mortality (PCA2 × *APOE2*: − 0.73 ± 0.31*, p* = 0.019). In conclusion, greater executive function/visuo-spatial abilities were associated with reduced CVD-specific mortality, particularly among Whites. Greater “attention/working memory” coupled with higher *APOE2* dosage was linked with reduced all-cause and CVD mortality risks.

## Introduction

Poor cognitive function measured in mid-^1^ and later-life^2^ is associated with subsequent mortality risk but the mechanisms underlying these associations remain unclear. Cognitive function is likely the result of complex interactions between environmental and genetic influences over the life course that may also shape trajectories of health and mortality. Beyond the social and physical implications, changes in cognitive function may signal broader biological changes or genetic mechanisms that extend beyond neurodegenerative processes. For example, it is well established that the Apolipoprotein E (*APOE*) gene plays an important role in cognitive health^3^. Specifically, carriers of the ε4 allele face greater risk of cognitive decline and impairment including Alzheimer’s disease (AD) relative to non-carriers^4^ whereas carriers of the ε2 allele experience lower risk of AD and cognitive impairment, on average^5^. Studies have shown, for example, that *APOE* genotype affects amyloid-β (Aβ) metabolism as well as cholesterol homeostasis, neurovascular functions, and neuroinflammation which are believed to play important roles in AD pathology^6^. Moreover, as a major cholesterol carrier that supports lipid transport and injury repair in the brain, *APOE* has also been implicated for its role in cardiovascular disease (CVD) pathology^7^ and mortality^8,9^. In addition, and based on a meta-analysis of > 60 studies, cognitive impairment, including overt dementia, has been linked to increased risk of all-cause mortality^10^. However, the extent to which *APOE* may moderate the association between cognitive performance and mortality remains unclear. Prior work that has examined the interplay between *APOE*, cognitive performance, and mortality has typically drawn conclusions based on European ancestry populations^8,11–18^. While most of these studies focused on either *APOE* genotype’s association with mortality or cognitive performance, a few have examined interactive associations, suggesting that the association between cognitive performance and mortality may indeed be modulated by *APOE2* or *APOE4* status or dosage. In light of the well-known wide black-white disparities in all-cause and cause-specific mortality^19,20^ coupled with differences in *APOE* genotypic frequency between Whites and African Americans^12,21,22^ and the differential effect of *APOE* dosage on various cognitive outcomes over time by race^12,22,23^, it is plausible that these relationships vary with respect to race. More specifically, *APOE4* and *APOE2* dosages were shown in several studies to differ in distribution between Whites and African Americans, whereby the *ε3/ε3* genotype is more prevalent among Whites, while both *ε2* and *ε4* alleles occur at a greater frequency among African Americans^12,21,22^. In addition, a recent study has shown that in fact, *APOE4* dosages are associated with cognitive decline, specifically in the domain of verbal memory, only among Whites, with inconsistent findings observed among African Americans^22^, therefore replicating previous findings within a cohort of White middle-aged adults participating the Baltimore Longitudinal Study of Aging (BLSA)^24^. Moreover, another study among Whites in the BLSA has shown that *APOE4* carrier status increased all-cause mortality risk in men and interacted with time-dependent AD to increase the risk of this outcome^18^. Nevertheless, whether relationships among *APOE* genotype, cognition and mortality are consistent between Whites and African Americans in a racially and socio-economically diverse cohort of urban adults remains unclear.

The objectives of this work were to (i) characterize the associations between *APOE* genotypes and all-cause and CVD-specific mortality in a racially and socio-economically diverse cohort of urban White and African American middle-aged adults, overall and across racial groups (ii) characterize the associations of cognitive performance and all-cause and CVD-specific mortality in the same cohort, overall and across racial groups (iii) investigate interactive associations of cognitive performance and *APOE* genotypes in relation to all-cause and CVD mortality, overall. It is hypothesized that *APOE4* and *APOE2* dosages would increase and decrease mortality risk, respectively, particularly among Whites and that poor cognitive performance is predictive of mortality, perhaps differentially by race. Finally, it is expected that *APOE2* and higher cognitive performance may act in synergy to reduce mortality risk, whereas *APOE4* and poorer cognitive performance may act in synergy to increase mortality risk.

## Subjects and methods

### Database

We used data from the Healthy Aging in Neighborhoods of Diversity across the Life Span (HANDLS), a prospective, community-based longitudinal cohort study of 3720 socioeconomically diverse African American and White men and women aged 30–64 years at baseline in 2004^25^. Participants were recruited from select communities in Baltimore, Maryland. Baseline data collection was conducted in two phases. For Visit 1 (2004–2009), HANDLS researchers conducted household interviews in participants’ homes (Phase 1). Phase 2 involved physical examinations which took place in Medical Research Vehicles (MRVs) parked in participants’ neighborhoods. The first wave of follow-up was conducted approximately five years later (Visit 2; 2009–2013) in which all examinations were performed on MRVs. HANDLS was approved by the Institutional Review Board of the National Institute of Environmental Health Sciences. All participants provided written informed consent. Details on study protocols and sample ascertainment have been described elsewhere^25^. Data are available upon request to researchers with valid proposals who agree to the confidentiality agreement as required by our Institutional Review Board. We publicize our policies on our website https://handls.nih.gov. All analyses were performed in accordance with relevant institutional guidelines and regulations.

### Study subjects

We set distinct inclusion criteria for our analyses as reflected in Fig. S1 (Sample 1 to 3), with gradual exclusion of participants with missing data on *APOE* genotype and/or cognitive performance data. Figure S1 also shows that among 3,720 participants recruited at baseline, we excluded 780–1087 individuals with missing or non-credible cognitive test information at both visits (mainly due to physical limitations, low literacy and other causes), resulting in an analytic sample between 2654 and 2922 depending on the cognitive test. These samples were only used to estimate baseline performance on cognitive tests, using up to two waves of data.

From this sample, we excluded participants who died within one year of their baseline interview (n = 13–17) and further excluded 338–645 individuals with missing or non-credible cognitive test information at both visits for at least one test score. This resulted in an analytic sample of 2260 individuals indicated in Fig. S1 as Sample 1. This sample was used to examine the association between cognitive performance and mortality outcomes.

To generate our inclusion sample for analyses that included *APOE* genotype, we began with the 3720 participants recruited at baseline and excluded decedents who died within one year of their baseline interview (n = 35); and we excluded 1339 individuals without information on *APOE* genotype yielding an analytic sample of 2346 individuals as shown in Fig. S1 (Sample 2). We used Sample 2 to evaluate the relationship between *APOE* genotype and mortality.

From Sample 2, as shown in Fig. S1, we excluded 576 individuals with missing or non-credible cognitive test information at both visit for all tests. This resulted in an analytic sample of 1770 individuals hereafter referred to as Sample 3 in Fig. S1. This sample was used to test interactions between *APOE* genotype and cognitive performance in relation to mortality outcomes.

### Mortality outcomes

Mortality status in the HANDLS was obtained through linkages to the National Death Index (NDI), National Center for Health Statistics. Information about underlying cause of death was obtained from death certificates and classified in accordance with the International Statistical Classification of Diseases, Version 10 (ICD-10). Deaths attributed to CVD included CVD-related diagnosis codes (ICD-10 codes I00-99.9) listed as the underlying or contributing cause of death, respectively defined by the US Department of Health and Human Services and the Centers for Disease Control and Prevention as “the disease or injury that initiated the chain of morbid events that led directly and inevitably to death” and “all other significant diseases, conditions, or injuries that contributed to death but which did not result in the underlying cause of death”^26^. Vital status information for all participants is available from enrollment (2004–2009) through December 31st, 2018 (last date of death available).

### Exposures

#### Cognitive functioning

HANDLS investigators conducted detailed cognitive assessments which included the Mini-Mental State Examination (MMSE), the California Verbal Learning Test (CVLT) immediate (List A) and Delayed Free Recall (DFR), Digit Span Forward and Backwards tests (DS-F and DS-B), the Benton Visual Retention Test (BVRT), Animal Fluency test (AF), Brief Test of Attention (BTA), Trails A and B, and the Clock Drawing Test (CDT) (Method S1). Empirical bayes estimators of baseline performance were predicted from mixed-effects linear regression models. These models assumed missingness at random. The empirical bayes estimators were highly correlated with their corresponding raw baseline performance in a complete case analysis, strengthening their validity and the assumption for missingness at random. We used principal components analysis to reduce the later 10 of these test scores, estimated from mixed-effects linear regression models (i.e. empirical bayes estimators for baseline cognitive performance), and generate uncorrelated principal components (PCs, Methods S2–S3; Tables S2-S3), scaled as a z-score with a greater score indicating better performance at baseline. This procedure resulted in three PCs. MMSE baseline performance was also estimated using mixed-effects linear regression model using the total MMSE score.

#### APOE genotype

*APOE* genotype is comprised of two variants (rs429358 [APOE-C112R] and rs7412 [APOE-R158C]) which results in three common isoforms: *APOE* ε2, ε3 and ε4^27^. These variants were genotyped using Taqman Assays (Applied Biosystems Assay-On-Demand part numbers C__3084793_20 and C__904973_10) on a 7900HT Sequence Detection System (Applied Biosystems).Absolute quantification was performed in four stages on ThermoHybaidPCR cycler blocks after which each polymerase chain reaction plate was held at 4 °C until returning to the 7900HT Sequence Detection System to conduct allelic discrimination^27^. We focused on the ε4 and ε2 allele dosages (0, 1 or 2) in our main analysis, while the genotype was examined only for descriptive purposes, with ε3/ε3 being considered as the common referent category.

#### Covariates

We adjusted for several potential confounders [all measured at the baseline visit, v_1_ (2004–2009)] in the relationship between cognitive performance and mortality. Covariates included age (continuous, years), sex (male, female), self-identified race (White, African American), educational attainment (less than high school [HS], HS, more than HS), poverty status (below vs. above 125% the federal poverty line), categorized as such by using the US Census Bureau poverty thresholds for 2004^28^ relying on income, total family size including children under age 18 years (found in the household though not selected in HANDLS), and literacy (Wide Range Achievement Test, third edition [WRAT-3]). Measures of health and lifestyle included a binary indicator of illicit drug use (marijuana, opiates, and cocaine), smoking status (never or former smoker, active smoker), body mass index (BMI, continuous), self-rated health (poor/average, good, very good/excellent), diet quality assessed using the Healthy Eating Index 2010 (HEI-2010)^29^, total caloric intake, and depressive symptoms assessed using the 20-item Center for Epidemiological Studies-Depression scale (CES-D). In addition, we accounted for comorbidities which included hypertension, diabetes, dyslipidemia, CVD, and a co-morbidity index which was calculated as an unweighted sum of the following conditions: hypertension (yes [1], no [0]), diabetes (diabetic [2], pre-diabetic [1], not diabetic [0]) and dyslipidemia or statin use (yes [1], no [0]), and self-reported history of CVD (yes [1], no [0]), which included atrial fibrillation, angina, coronary artery disease, congestive heart failure, and myocardial infarction. Thus, the index could range between 0 and 5. Multiple imputation (k = 5 imputations with 10 iterations) of covariates using chained equations was applied to obtain the largest sample available with complete exposure and outcome data. In addition, other variables were used for sensitivity analysis, namely total and HDL-Cholesterol, with measurement methodology and criteria used described elsewhere^30,31^.

### Statistical analysis

We used Stata release 16^32^ to conduct all analyses. We characterized the overall analytic sample at baseline using means and proportions; t-tests were used to examine racial differences in baseline characteristics. We used a series of regression models to evaluate whether baseline characteristics varied by race. To examine the association between cognitive performance and all-cause and CVD-specific mortality, we estimated a series of Cox proportional hazard regression models with sequential covariate adjustment. Age (years) on study was used as the underlying time scale. Baseline age was used as time at entry in all analyses (i.e., delayed entry).

First, we fit a series of models evaluating associations between all-cause and CVD-specific mortality and each of MMSE (A), PCA1 (B), PCA2 (C), and PCA3 (D) with adjustment for age, sex, race, poverty status, WRAT-3, and the inverse mills ratio (Models 1A–1D: Sample 1, Fig. S1). We then fit a series of models with additional adjustment for the remaining aforementioned covariates (Models 2A-2D, also using Sample 1, Fig. S1); none of these models included *APOE* genotype. After fitting a model for the overall sample, we stratified our sample by race and repeated our analyses to examine race-specific pathways. We followed a similar process to examine the association between *APOE* genotype and all-cause and CVD-specific mortality in the overall sample. Specifically, we evaluated mortality risk by the number of *APOE* ε4 alleles (0, 1 or 2) [(Models 1A (minimal adjustment) and 2A (full covariate adjustment model); Sample 2, Fig. S1]. We separately examined associations between the number of *APOE* ε2 alleles (0, 1 or 2) and mortality using the same approach. These models did not account for cognitive performance. To test for a moderating effect of *APOE* genotype on the association between cognitive performance and mortality, we fit a series of models that separately examined associations between one of four cognitive performance metrics and mortality with an interaction term for *APOE2* or *APOE4* allelic dose (Fig. S1). This analysis was carried out among individuals comprising Sample 3 in (Fig. S1). Race-stratified analyses were not conducted due to limited statistical power among this smaller sample. For brevity, we only report results from fully adjusted models in the paper. Predicted smoothed hazard rates and Kaplan-Meir estimates were presented to visualize key findings, by categorizing continuous cognitive measures (e.g. PCAs) into tertiles and stratifying by *APOE2* or *APOE4* doses and/or by race. X^2^ test and Χ^2^ test for trend comparing observed and expected events with associated p-values were also used to compare survival probability estimates. Additionally, we evaluated associations between total and high-density lipoprotein (HDL) allostatic load markers (AL) at v_1_ (2004–2009) and *APOE2* or *APOE4* dosages. Finally, we ran the interaction models again with adjustment for total and HDL AL markers. Those two markers were evaluated using previously published criteria for elevated total cholesterol and reduced HDL-cholesterol to construct the AL overall measure^31^.

We accounted for sample selection using a two-stage Heckman selection strategy in the Cox proportional hazards models. In stage 1, we used a probit model to regress an indicator of sample inclusion on baseline age, sex, race, and poverty status which provided an Inverse Mills Ratio (IMR). In Stage 2, we estimated the Cox proportional hazards regression models with adjustment for the IMR and the aforementioned confounders^33^.

We decided a priori to set the Type I error rate for the main effect and interaction terms prior to correcting for multiplicity to 0.05 and 0.10, respectively^34^. We accounted for outcome multiplicity (i.e., all-cause and CVD-specific mortality) using a familywise Bonferroni approach. Thus, we tested distinct hypotheses pertaining to associations between each cognitive domain and *APOE* genotype. In this context, we adjusted significance levels for main effects to *p* < 0.025 (0.05/2). Significance levels for the two-way interaction terms were adjusted to 0.10/2 = 0.05. This approach is similar to previously published work^35^.

### Disclaimer

The views expressed in this article are those of the authors and do not reflect the official policy of Fort Belvoir Community Hospital, the Defense Health Agency, Department of Defense, or the U.S. Government. Any discussion or mention of commercial products or brand names does not imply or support any endorsement by the Federal Government.

## Results

### Characteristics of study participants by race

Study sample characteristics are presented in Tables 1 and S1 across race groups, using the smallest analytic sample (Sample 3, Fig. S1). Overall, 80.5% of participants were non-carriers for ε2 (80.5%) and 66.7% were non-carriers for ε4 alleles. Compared with Whites, African Americans were more likely to be carriers of an ε4 allele. The ε2 allele was also more prevalent among African Americans. Among Whites, the ε3/ε3 genotype was more prevalent compared with African Americans (61.1% vs. 43.0%, p < 0.05).

**Table 1:** Study sample characteristics, overall and by race for sub-sample with complete and valid cognitive performance data at either visit and *APOE* genotype data, excluding participants who died during first year, HANDLS 2004–2013.

	Overall	Whites	African American
	(X ± SE), %	(X ± SE), %	(X ± SE), %
	(N = 1770)	(N = 794)	(N = 976)
***APOE2* allelic dose**			
0	80.5 ± 0.9	84.5 ± 1.3	77.2 ± 1.3
1	18.8 ± 0.9^b^***	14.9 ± 1.3	21.9 ± 1.3
2	0.7 ± 0.2	0.5 ± 0.3	0.9 ± 0.3
**X ± SE**			
* APOE4* allelic dose			
0	66.7 ± 1.1	74.2 ± 1.6	60.7 ± 1.6
1	29.4 ± 1.1^b^***	23.6 ± 1.5	34.1 ± 1.5
2	3.9 ± 0.5^b^***	2.3 ± 0.5	5.2 ± 0.7
**X ± SE**			
Sex, % male	42.8 ± 1.2	43.7 ± 1.8	42.1 ± 1.6
Age at v_1_, years	48.496 ± 0.218	48.606 ± 0.325	48.406 ± 0.294
African American, %	55.1 ± 1.2	0.000	100.0
Poverty status, % (< 125% federal poverty line)	38.9 ± 1.2***	31.0 ± 1.6	45.3 ± 1.6
**Education, completed, %**			
< HS	5.9 ± 0.6^b^***	8.5 ± 1.0	3.9 ± 0.6
HS	59.1 ± 1.2	55.9 ± 1.8	61.9 ± 1.6
> HS	35.0 ± 1.1	35.7 ± 1.7	34.2 ± 1.5
Literacy, WRAT-3 score	42.593 ± 0.185^b^***	44.810 ± 0.274	40.789 ± 0.236
MMSE, baseline performance^a^	27.75 ± 0.04^b^***	28.10 ± 0.06	27.47 ± 0.05
PCA1 (verbal memory/fluency)^a^	+ 0.026 ± 0.035^b^***	+ 0.416 ± 0.056	− 0.291 ± 0.043
PCA2 (attention/working memory)^a^	+ 0.034 ± 0.034^b^***	+ 0.399 ± 0.052	− 0.262 ± 0.041
PCA3 (executive function/visuo-spatial)^a^	+ 0.027 ± 0.032^b^***	+ 0.354 ± 0.040	− 0.239 ± 0.047

Values are means (X) ± SE for continuous variables and % for categorical variables. The sample selected has complete data on MMSE and 10 other cognitive test scores at visits 1 and/or 2 and complete data on APOE genotypes. Aside from cognitive measures which were predicted at v_1_ using v_1_/v_2_ observations, all other measures presented in this Table are v_1_ measures (2004–2009).

*APOE* Apolipoprotein E genotype, *HANDLS* Healthy Aging in Neighborhood of Diversity across the Lifespan, *HS* High school, *MMSE* Mini-Mental State Examination, *PCA* Principal Components Analysis, *X* mean, *WRAT-3* Wide Range Achievement Test, 3rd revision.

^a^MMSE baseline performance, empirical bayes estimator from mixed-effects linear regression model (See Method S2). PCA1 through PCA3 are the principal components, rotated using varimax rotation, extracted from 10 cognitive performance test score empirical bayes estimator for baseline performance, excluding MMSE (See Method S3).

^b^p < 0.05 upon further adjustment for age, sex, and poverty status in multiple linear, logistic and multinomial logit models with race entered as the main predictor.

*p < 0.05** *p* < 0.01; *** *p* < 0.001, *t*-test for null hypothesis of no between-race differences.

V1 cognitive performance test scores indicated poorer performance among African Americans compared with Whites, including in terms of global mental status, as measured by the MMSE. This was translated also into lower PCA1 (verbal memory/fluency), PCA2 (attention/working memory) and PCA3 (executive function/visuo-spatial) scores reflecting poorer performance on all three domains of cognition among African Americans compared with Whites, as well as lower empirical bayes estimated baseline performance on MMSE, even upon adjustment for age, sex and poverty status in a linear regression model. Other results are detailed in Results S1.

### Associations of cognitive performance and APOE genotype with all-cause and CVD mortality

The 2,260 individuals comprising Sample 1 contributed 25,632 person-years of follow-up (median [minimum, maximum] length of follow-up, 11.6 [1.1, 14.3] years) during which time there were 340 deaths overall (crude death rate: 13.3/1,000 person-years) and 99 CVD-specific deaths (crude death rate [CDR]: 3.9/1,000 person-years). In Sample 2, which comprised 2,346 individuals, there were a total of 382 deaths over 26,364 person-years of follow-up (median [minimum, maximum] length of follow-up, 11.5 [1.0, 14.4] years) yielding a CDR of 10.7/1000 person-years. There were 113 CVD-specific deaths (CDR: 4.3/1,000 person-years). In Sample 3, which was restricted to 1,770 individuals with *APOE* genotype information and complete cognitive test information at both visits for all tests, there were a total of 260 deaths (CDR: 13.0/1,000 person-years)—76 of which were attributable to CVD (CDR: 3.8/1,000 person-years)—over 19,963 person-years of follow-up (median [minimum, maximum] length of follow-up, 11.5 [1.1, 14.4] years).

Table 2 presents associations of cognitive performance scores (MMSE, PCA1 through PCA3) with all-cause and CVD mortality, overall and by race, using a two-step modeling process. In the minimally adjusted models (Model 1A-1D), only PCA3 reflecting poorer baseline performance on domains of executive function/visuo-spatial domains (z-score) was linked with lower all-cause (HR = 0.93; 95% CI 0.87–0.99, p = 0.030) and CVD mortality risk (HR = 0.85, 95% CI 0.77–0.95, p = 0.001). The latter association was particularly stronger among Whites (HR = 0.62, 95% CI 0.48–0.80, p < 0.001), compared with African Americans (HR = 0.89, 95% CI 0.90–1.01, p = 0.067). After correcting for multiple testing, only associations among Whites remained statistically significant. Adding a race × PCA3 interaction term in the unstratified model, indicated that race was a significant effect modifier in the case of CVD (p = 0.005 for race × PCA3 in model 1D) but not all-cause mortality (P = 0.11 for race × PCA3 in model 1D). In models 2A-2D, further adjustment for lifestyle and health-related factors resulted in marked attenuation of those associations, although they remained statistically significant at a type I error of 0.05, with a stronger putative protective effect found among Whites with respect to PCA3 vs. CVD mortality (HR = 0.70, 95% CI 0.51–0.95, p = 0.023).

**Table 2:** Association of cognitive performance with all-cause and CVD mortality: overall and race-specific Cox Proportional hazards models: HANDLS 2004–2018.

	Overall		Whites		African Americans	
	(N = 2260)		(N = 948)		(N = 1312)	
	HR	95% CI	HR	95%CI	HR	95% CI
**Models 1A–1D**						
All-cause mortality	n = 340 deaths		n = 129 deaths		n = 211 deaths	
MMSE	0.98	0.91, 1.06	0.91	0.80, 1.03	1.01	0.92, 1.11
PCA1 (verbal memory/fluency)	0.96	0.88, 1.05	0.92	0.81, 1.06	0.99	0.88, 1.11
PCA2 (attention/working memory)	0.98	0.89, 1.08	1.02	0.87, 1.20	0.96	0.85, 1.09
PCA3 (executive function/visuo-spatial)	0.93*	0.87, 0.99	0.80**	0.68, 0.93	0.95	0.88, 1.03
CVD mortality	n = 99 deaths		n = 33 deaths		n = 66 deaths	
MMSE	0.96	0.83, 1.10	0.82	0.65, 1.03	1.04	0.87, 1.24
PCA1 (verbal memory/fluency)	0.90	0.77, 1.07	0.87	0.66, 1.16	0.96	0.78, 1.18
PCA2 (attention/working memory)	0.84	0.70, 1.02	0.97	0.70, 1.36	0.80	0.63, 1.02
PCA3 (executive function/visuo-spatial)	0.85^a^***	0.77, 0.93	0.62***	0.48, 0.80	0.89	0.80, 1.01
**Models 2A–2D**						
All-cause mortality	n = 340 deaths		n = 129 deaths		n = 211 deaths	
MMSE	0.99	0.92, 1.07	0.95	0.83, 1.08	1.01	0.91, 1.11
PCA1 (verbal memory/fluency)	0.99	0.91, 1.09	0.99	0.85, 1.14	1.01	0.90, 1.14
PCA2 (attention/working memory)	0.98	0.89, 1.09	1.02	0.86, 1.21	0.96	0.84, 1.09
PCA3 (executive function/visuo-spatial)	0.95	0.88, 1.02	0.86	0.72, 1.03	0.95	0.87, 1.04
CVD mortality	n = 99 deaths		n = 33 deaths		n = 66 deaths	
MMSE	0.97	0.85, 1.12	0.90	0.71, 1.14	1.03	0.86, 1.23
PCA1 (verbal memory/fluency)	0.95	0.80, 1.13	1.00	0.73, 1.37	0.99	0.80, 1.23
PCA2 (attention/working memory)	0.88	0.72, 1.06	1.09	0.77, 1.54	0.82	0.64, 1.04
PCA3 (executive function/visuo-spatial)	0.85^a^**	0.77, 0.95	0.70*	0.51, 0.95	0.88*	0.77, 1.00

Models 1A-1D included each of 4 cognitive performance variables separately as the main predictor for all-cause or CVD mortality. The models were carried out in the overall population and stratified by race. These models adjusted only for age, sex, race, poverty status, education, the WRAT-3 score, and the inverse mills ratio using imputed data. Models 2A-2D followed a similar approach but adjusted further for all other lifestyle and health-related factors, namely current drug use, current tobacco use, body mass index, self-rated health, co-morbidity index, HEI-2010, total energy intake, and the CES-D total score.

*CVD* Cardiovascular Disease, *HANDLS* Healthy Aging in Neighborhood of Diversity across the Lifespan, *HS* High school, *MMSE* Mini-Mental State Examination, *PCA* Principal components analysis, *WRAT-3* Wide Range Achievement Test, 3rd revision.

PCA1 through PCA3 are the principal components, rotated using varimax rotation, extracted from 10 cognitive test score baseline performance, excluding MMSE.

^a^p < 0.05 for Race × cognitive performance interaction in models that are unstratified by race to which this 2-way interaction was included.

*p < 0.05**p < 0.01; ***p < 0.001, *t*-test for null hypothesis of Log_e_(HR) = 0.

Table 3 presents results of Cox proportional hazards models with outcome being ages to all-cause and CVD mortality, and exposures being alternatively, *APOE2* and *APOE4* dosages in minimally adjusted and fully adjusted models. In models adjusted only for main socio-demographic variables, education and literacy (Models 1A-1B), *APOE4* was associated with greater risk of CVD mortality (HR = 1.37, 95% CI 1.01–1.86, p = 0.041), an association that did not survive adjustment for multiple testing (p > 0.025). This association is shown graphically in Fig. S2 which displays smoothed hazard rates for CVD mortality by *APOE4* dose (p-trend = 0.031). This association did not differ significantly by race. A notable trend was also observed with respect to *APOE2* dose and CVD mortality (HR = 0.60, 95% CI 0.35–1.03, p = 0.065) in the overall population. Nevertheless, all these associations were markedly attenuated in fully adjusted models (p > 0.05).

**Table 3:** Associations of *APOE4* and *APOE2* doses with all-cause and CVD mortality: overall and race-specific Cox proportional hazards models: HANDLS 2004–2018.

	Overall		Whites		African Americans	
	(N = 2346)		(N = 1043)		(N = 1303)	
	HR	95% CI	HR	95%CI	HR	95% CI
**Models 1A–1B**						
All-cause mortality	n = 382 deaths		n = 153 deaths		n = 229 deaths	
*APOE4* dosage (0,1,2)	1.03	0.86, 1.24	0.95	0.69, 1.32	1.07	0.86, 1.34
*APOE2* dosage (0,1,2)	0.91	0.71, 1.17	0.98	0.64, 1.49	0.87	0.63, 1.19
CVD mortality	n = 113 deaths		n = 36 deaths		n = 77 deaths	
*APOE4* dosage (0,1,2)	1.37*	1.01, 1.86	1.67	0.97, 2.87	1.25	0.87, 1.79
*APOE2* dosage (0,1,2)	0.60	0.35, 1.03	0.60	0.21, 1.67	0.60	0.31, 1.14
**Models 2A–2B**						
All-cause mortality	n = 382 deaths		n = 153 deaths		n = 229 deaths	
*APOE4* dosage (0,1,2)	1.03	0.86, 1.23	0.87	0.63, 1.22	1.11	0.89, 1.39
*APOE2* dosage (0,1,2)	0.92	0.72, 1.19	0.94	0.62, 1.45	0.87	0.63, 1.20
CVD mortality	n = 113 deaths		n = 36 deaths		n = 77 deaths	
*APOE4* dosage (0,1,2)	1.33	0.98, 1.81	1.47	0.85, 2.56	1.27	0.88, 1.85
*APOE2* dosage (0,1,2)	0.61	0.35, 1.05	0.64	0.23, 1.82	0.59	0.30, 1.13

Models 1A–1B included *APOE4* or *APOE2* doses alternatively as the main predictor for all-cause or CVD mortality. The models were carried out in the overall population and stratified by race. These models adjusted only for age, sex, race, poverty status, education, the WRAT-3 score, and the inverse mills ratio using imputed data. Models 2A-2B followed a similar approach but adjusted further for all other lifestyle and health-related factors, namely current drug use, current tobacco use, body mass index, self-rated health, co-morbidity index, HEI-2010, total energy intake, and the CES-D total score.

*APOE* Apolipoprotein E gene, *BMI* Body Mass Index, *CES-D* Center of Epidemiological Studies-Depression, *CVD* Cardiovascular Disease, *HANDLS* Healthy Aging in Neighborhood of Diversity across the Lifespan, *HEI-2010* Healthy Eating Index, 2010, *HS* High school, *MMSE* Mini-Mental State Examination, *WRAT-3* Wide Range Achievement Test, 3rd revision.

*p < 0.05**p < 0.01; ***p < 0.001, *t*-test for null hypothesis of Log_e_(HR) = 0.

Tables 4 and S4 show a series of Cox proportional hazards models which examines interactions between each of 4 cognitive performance scores (MMSE, PCA1 through PCA3) with *APOE2* and *APOE4* doses in relation to all-cause and CVD mortality, presenting only fully-adjusted models in the total sample, for simplicity. Our findings indicate that PCA2 interacted synergistically with *APOE2* dosage to reduce the risk of all-cause (PCA2 × *APOE2*: − 0.33 ± 0.13, *p* = 0.010) and CVD mortality (PCA2 × *APOE2*: − 0.73 ± 0.31, *p* = 0.019). In both outcomes and within Model 3A, only the *APOE2* × PCA2 remained statistically significant after adjustment for multiple testing, indicating super-multiplicative effect of a higher PCA2 and a greater *APOE2* dose on the reduction in all-cause and CVD mortality risk. PCA2 score reflected better performance on the attention and working memory domains.

**Table 4:** Interactive associations of cognitive performance and *APOE2* dosage with all-cause and CVD mortality in the overall sample, Cox proportional hazards models: HANDLS 2004–2018.

	All-cause mortality		CVD mortality	
	(N = 1770)		(N = 1770)	
	n = 260 deaths		n = 76 deaths	
	Log_e_(HR)	(SE)	Log_e_(HR)	(SE)
**Models 1A: MMSE interaction with *APOE2***				
MMSE	− 0.01	0.05	0.06	0.09
*APOE2*	3.52	2.13	3.09	4.42
MMSE × *APOE2*	− 0.13	0.08	− 0.13	0.16
**Models 2A: verbal memory/fluency domains (**PCA1): **interaction with *APOE2***				
PCA1	0.02	0.06	0.00	0.11
*APOE2*	− 0.09	0.16	− 0.54	0.35
PCA1 × *APOE2*	0.06	0.11	0.06	0.26
**Models 3A: Attention and working memory domains (**PCA2): **interaction with *APOE2***				
PCA2	0.13*	0.06	0.04	0.11
*APOE2*	− 0.22	0.17	− 1.02*	0.45
PCA2 × *APOE2*	− 0.33**	0.13	− 0.73*	0.31
**Models 4A: Executive function and visuo-spatial domains (**PCA3): **interaction with *APOE2***				
PCA3	− 0.04	0.05	− 0.10	0.07
*APOE2*	− 0.12	0.16	− 0.66	0.37
PCA3 × *APOE2*	− 0.11	0.10	− 0.21	0.13

Models included each of 4 cognitive performance variables separately as the main predictor for all-cause or CVD mortality and interacted this main predictor with *APOE2* dosage. The models were carried out in the overall population only. All models adjusted only for age, sex, race, poverty status, education and the WRAT-3 score using imputed data, in addition to other lifestyle and health-related factors, namely current drug use, current tobacco use, body mass index, self-rated health, co-morbidity index, HEI-2010, total energy intake, CES-D total score, and the inverse mills ratio.

*APOE* Apolipoprotein E gene, *BMI* Body Mass Index, *CES-D* Center of Epidemiological Studies-Depression, *CVD* Cardiovascular Disease, *HANDLS* Healthy Aging in Neighborhood of Diversity across the Lifespan, *HEI-2010* Healthy Eating Index, 2010, *HS* High school, *MMSE* Mini-Mental State Examination, *PCA* Principal Components Analysis, *WRAT-3* Wide Range Achievement Test, 3rd revision.

*p < 0.05; **p < 0.01; ***p < 0.001, *t*-test for null hypothesis of Log_e_(HR) = 0.

In Table S5, we present the associations between *APOE* genotype and total as well as HDL cholesterol AL markers. *APOE2* was significantly negatively associated with both the total and HDL cholesterol AL markers, whereas *APOE4* was significantly positively associated with these two markers. Tables S6–S7 display results that extend our findings presented in Tables 4 and S4 by including additional adjustments for total as well as HDL cholesterol AL markers. However, the results were unchanged, and neither total nor HDL cholesterol AL markers were associated with mortality outcomes in our interaction analyses.

**Figure 1:**
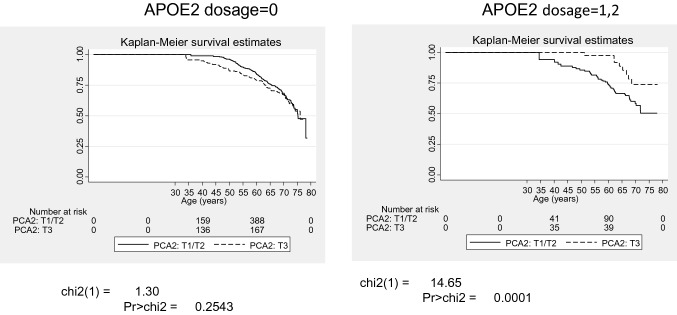
Kaplan–Meir survival estimates for all-cause mortality by *APOE2* dosage (0 vs. 1/2) and tertiles of cognitive performance in attention and working memory domains (T1/T2 vs. T3): HANDLS 2004–2018. *APOE* Apolipoprotein E, *HANDLS* Healthy Aging in Neighborhoods of Diversity Across the Life Span; T1–T3 Tertiles.

A key finding is illustrated in Fig. 1 which displays Kaplan-Meir survival estimates for all-cause mortality by PCA2 tertiles and stratified by *APOE2* status. This figure shows that PCA2 is predictive of lower all-cause mortality only in the group with at least one *APOE* ε2 allele (p < 0.001), illustrating the interaction between PCA2 and *APOE2*, the combination of which is linked to lower all-cause mortality.

## Discussion

To our knowledge, this study is among few to systematically examine associations between *APOE* genotype, cognitive performance and mortality, and the first to test those associations in a racially diverse sample of urban middle-aged adults. Higher *APOE4* dose was associated with increased CVD-mortality (hazard ratio [HR] per allele = 1.37; 95% CI 1.01–1.86, p = 0.041); *APOE2* dosage’s association with CVD-mortality was non-significant (HR = 0.60; 95% CI 0.35–1.03, p = 0.065). On the other hand, better performances on domains of executive function/visuo-spatial was linked to lower risk of all-cause and CVD mortality, the latter being more pronounced among Whites. We also observed an interaction of a better performance on the attention/working memory domain and a greater *APOE2* dose on the reduction in all-cause and CVD mortality risk.

Poor cognitive function is associated with functional dependence^36^ and poor quality of life^37^ which are independently associated with mortality^38,39^. Moreover, cognitive health is associated with increased rates of comorbid conditions^40^ and hospitalizations^41^. *APOE4* dosage or status have been linked to increased risk for all-cause and cardiovascular mortality in a number of studies^8,11–15,18^; while a protective effect of *APOE2* dosage or status was found in relation to all-cause but mostly cardiovascular mortality^8,12,13,16,17^. This genotype-mortality association was not detected in other studies for *APOE2*^11,42^ and for *APOE4* dosages^42^. It is worth noting, however, that most of these studies were conducted among individuals of EA. Our study included both White and African American middle-aged urban adults, and we detected associations of *APOE4* dosage with cardiovascular mortality, in the total sample and mainly in the minimally adjusted model. Only a trend suggestive of a protective effect of *APOE2* dosage was detected in the case of cardiovascular mortality in our sample. However, for both *APOE4* an *APOE2* dosages, those associations were attenuated by further adjustment for lifestyle and health-related factors, many of which may be mediating these relationships. Also worth of noting is that this analysis (i.e. *APOE* dosage vs. mortality) was carried out on a larger sample than that of *APOE* dosages, cognitive performance and mortality analyses. Thus, we were not able to directly test mediation effects between *APOE* dosage and mortality through cognitive performance without significant loss in statistical power. Such loss in statistical power would particularly impact the significance of the total effect of *APOE4* on CVD mortality in the smaller sample (N = 1770) as opposed to the larger sample (N = 2346).

Furthermore, our study indicated that there was an interaction of a better performance on the attention/working memory domain and a greater *APOE2* dosage on the reduction in all-cause and CVD mortality risk. This is an important finding indicating that *APOE2* dosage may act in synergism with better performance on specific cognitive domains to improve survival, particularly by reducing CVD-mortality. Previous studies examining this research question of interaction between *APOE* genotype and cognition in relation to mortality are scarce and none have examined this among African American or racially diverse populations. Nevertheless, numerous studies have shown that poor cognitive performance, as well as dementia occurrence are predictive of higher mortality risk^18,43–46^. In our study, we found that overall, better performances on domains of executive function/visuo-spatial was linked to lower risk of all-cause and CVD mortality, the latter being more pronounced among Whites. This association may be due to higher variability in cognitive performance among Whites compared with African Americans, and therefore greater ability to detect such an association. One of few studies that systematically examined interactive associations concluded that *APOE4* carrier status increases all-cause and cardiovascular mortality risks and interacted with sex and time-dependent AD status to affect all-cause mortality in a sample of White adults living in Baltimore city^18^. More specifically, there was synergism between time-dependent AD and *APOE4* carrier status in relation to all-cause mortality among men^18^. Our findings are in line with this study in two aspects: First, we found that the relationship between cognitive performance and mortality was stronger among Whites. Second, we found that there was synergism between *APOE2* dosages and better performance in several domains of cognition (“attention/working memory”) with reduced risk of mortality, in the overall population. However, statistical power was limited to study interactive associations by race. Pending randomized controlled trials, the association between executive function/visuo-spatial and reduced risk of CVD mortality among Whites implies that enhancing these domains of cognition in Whites may be added among preventive strategies reducing the burden of CVD mortality in comparable urban populations. However, such strategy may not be sufficient to reduce risk of CVD mortality among African Americans.

Although known for associations with CVD, dementia, and Alzheimer’s disease^47^, the most frequent *APOE* alleles (ε2, ε3, ε4) have also been linked to normal and pathological aspects of neurobiology at various developmental stages throughout the lifespan^48^ and may also influence epigenetic changes over time^49^. Evidence for an association of *APOE* genotype with cognitive function is less clear cut^50^, despite the fact that *APOE* ε4 may accelerate amyloid deposition^51^ and the impact of amyloid deposition on cognitive function may be modified by *APOE* ε4^52^. *APOE* is a major cholesterol carrier that supports lipid transport and injury repair in the brain^53^ and is thought to play an important role in modifying systemic and brain inflammatory responses^54,55^. The genetic variants that comprise *APOE* differ in their binding affinity to serum cholesterol which influences its ability to metabolize dietary fat in the blood^56^ such that carriers of *APOE2* tend to have lower total serum cholesterol whereas carriers of *APOE4* tend to have higher levels, on average, relative to *APOE3* homozygotes^56^, an association found also at younger ages^47^.

Our study has several strengths. Whereas prior work has focused almost exclusively on adults of European ancestry, our study is among the first to assess the link between cognition, *APOE* genotype, and all-cause and CVD- specific mortality among a diverse, urban sample of adult men and women. Additional strengths of the HANDLS study include the breadth of its cognitive battery and its extensive mortality follow-up, as well as its representativeness of numerous urban settings across the United States with comparable racial compositions^25^. Our analysis on cognitive performance used data-driven techniques, such as PCA, providing a realistic characterization of multidomain differences in participants cognitive functioning at the baseline visit. Moreover, we accounted for genetic sample selection and outcome multiplicity in statistical analysis.

Our study findings should be interpreted cautiously in light of several limitations. First, some assumptions were made which may or may not be true, including missingness at random for mixed-effects linear regression models and orthogonality of the principal components. Moreover, analysis on CVD mortality had lower statistical power to all-cause mortality, which may explain the findings with respect to *APOE* genotype and CVD mortality. Larger samples of middle-aged adults obtained from similar populations may have yielded more precise estimates. Finally, despite careful modeling, use of the largest available samples and of standardized methods to obtain various measures, residual confounding, measurement and selection biases cannot be completely ruled out.

In conclusion, with limited evidence of an association between *APOE2* or *APOE4* doses and CVD mortality risk, reduced all-cause and CVD mortality risks were predicted by better performance on domains of executive function/visuo-spatial performance, in particular among Whites, and by a synergistic interaction between *APOE2* dose and better performance on domains of attention/working memory. When we sequentially adjusted our interaction models for total and HDL cholesterol AL markers, the results were unchanged suggesting that the significant interactions may be explained by non-lipid related pathways. Future work that evaluates the role of other genetic variants, such as *ABCA7* for which the effect size on the relative odds of AD is significantly higher in African Americans^57^, among racially diverse samples is needed to advance our understanding of the pathways through which these genetic variants may contribute to disparities in AD pathology.

## Supplementary Information

Supplementary Information.
