# Supplementary material for: Associations between race, APOE genotype, cognition, and mortality among urban middle-aged white and African American adults
Source: Sci Rep. 2021 Oct 6;11:19849. doi: 10.1038/s41598-021-98117-2 (PMC8494809; doi:10.1038/s41598-021-98117-2)
Supplement: Supplementary file 1 — Supplementary Information. [file 41598_2021_98117_MOESM1_ESM.pdf]

ONLINE SUPPLEMENTAL MATERIALS

ASSOCIATIONS BETWEEN RACE, *APOE* GENOTYPE, COGNITION, AND MORTALITY AMONG URBAN MIDDLE-AGED WHITE AND AFRICAN AMERICAN ADULTS

Method S1: Description of cognitive tests, literacy and the CES-D

Mini-Mental State Examination (MMSE)

The MMSE [1] is a cognitive screener that captures global cognitive functioning by briefly measuring orientation, concentration, immediate and short-term memory, language and constructional praxis. Scores range from 0 to 30. Higher scores suggest better cognitive function.

California Verbal Learning Test (CVLT)

The CVLT [2] is a verbal learning and memory test that includes a 16-item word list. A modified version of the CVLT was used with three, as opposed to five, learning trials. Cued recall was not administered. To capture verbal learning and memory, CVLT outcomes variables were total correct score for List A (learning) and List A long-delay free recall (memory). The learning score ranged from 0 to 48 and the memory score ranged from 0 to 16. Higher scores indicate better verbal learning and memory. A more comprehensive description of CVLT can be found elsewhere [2].

Benton Visual Retention Test (BVRT)

The BVRT [3] is a measure of nonverbal memory and visuo-constructional abilities. Administration A, Form D was used. A modified error scoring system based off the BVRT manual was used to guide two trained examiners in scoring the BVRT. Resolution of discrepancies in scoring were attempted by the two examiners, however, if a consensus could not be achieved, MKT, a research psychologist, provided the score. The outcome variable was total errors, with higher values indicating lower visual memory scores.

Digit Span Forward and Backward (DS-F and DS-B)

The Wechsler Adult Intelligence Scale, Revised[4] Digit Span Forward and Backward primarily capture attention and working memory, a component of executive function. The tests were administered according to the manual's instructions. The outcome variable was the total score, which was the total number of correct answers for each test.

Category Fluency

Category fluency[5,6] is a measure of semantic verbal fluency, where participants are asked to generate as many animals as possible within a 60 second duration. Higher scores indicate better category fluency. The outcome variable was the total number of correctly generated words (i.e., words that were *not* intrusions and perseverations).

Brief Test of Attention (BTA)

For the BTA [7], a test of divided auditory attention, the examiner administered up to 10 trials of letters and numbers (4-18 items) that increased in length with each trial. Only the numbers portion of the test was administered. For each trial, participants were asked to disregard the number of letters read, while tracking how many numbers were recited. They were also told to keep their hands in fists to avoid finger counting. The outcome variable was the total number of correct trials.

Trail Making Tests A and B (TRAILS A and B)

The Trail Making Tests A and B[8] primarily capture attention and executive functioning, respectively. The main executive function subdomain that TRAILS B captures is set-shifting and cognitive control. Both trials also measure visuo-motor scanning and processing speed.

Participants were asked to draw a line between consecutive numbers (TRAILS A) and alternate between numbers and letters (TRAILS B) as quickly as they could. They were informed that they were being timed. The examiner pointed out errors that were then corrected by the participant. Errors were captured via increased time. Scores for TRAILS A and B reflected seconds to completion, where higher scores indicate poorer performance.

Clock Drawing Test – Clock to Command (CDT)

The Clock Drawing Test [9] is a measure of visuo-spatial abilities, that also captures elements of memory and executive function. Participants are instructed to draw a clock, put in all of the numbers, and set the hands to 10 minutes past 11. Performance is based off correct drawings of the clock face (0-2), numbers (0-4) and hands (0-4). Scores ranged from 0 to 10, with higher scores indicating better performance. Participants who did not score a perfect score on the command portion of the test were also asked to copy a clock with the hands set to 10 minutes after 11.

Wide Range Achievement Test – 3rd Edition: Word and Letter Reading Subtest (WRAT)

The WRAT Word and Letter Reading Subtest [10] is a test of reading ability that is often used as a proxy for literacy and quality of education. Participants were instructed to correctly read a list of 50 words that increased in difficulty. If the first five words were not correctly pronounced, letter reading was also administered. Standard instructions were used with the tan form. The outcome variable used was the total number of correctly pronounced words.

Center for Epidemiological Studies Depression Scale (CES-D)

The CES-D [11] is a 20-item measure of depressive symptomatology. Participants are asked to consider the frequency and severity of their symptoms over the last week. Scores ranged from

0 to 60. Scores of ≥16 indicated significant depressive symptoms and scores of ≥20 indicated a clinically significant amount of depressive symptoms.

Method S2: Mixed-effects regression models

The main multiple mixed-effects regression models can be summarized as follows:

	**Multi-level models** vs. **Composite models**		
**Eq. 1.1-1.4**	$Y_{ij} = \pi_{0i} + \pi_{1i}Time_{ij} + \varepsilon_{ij}$	$\pi_{0i} = \gamma_{00} + \gamma_{0a}X_{aij} + \sum_{k=1}^{l}\gamma_{0k}Z_{ik} + \zeta_{0i}$ $\pi_{1i} = \gamma_{10} + \gamma_{1a}X_{aij} + \sum_{m=1}^{n}\gamma_{1m}Z_{im} + \zeta_{1i}$	$Y_{ij} = \gamma_{00} + \gamma_{0a}X_{aij} + \sum_{k=1}^{l}\gamma_{0k}Z_{ik}$ $+ \gamma_{10}Time_{ij} + \gamma_{1a}X_{aij}Time_{ij}$ $+ \sum_{m=1}^{n}\gamma_{1m}Z_{im}Time_{ij}$ $+ (\zeta_{0i} + \zeta_{1i}Time_{ij} + \varepsilon_{ij})$

Where Y_{ij} is the outcome (Each cognitive test score measured at v_1 and/or v_2) for each individual "i" and visit "j"; π_{0i} is the level-1 intercept for individual i; π_{1i} is the level-1 slope for individual i; γ_{00} is the level-2 intercept of the random intercept π_{0i} ; γ_{10} is the level-2 intercept of the slope π_{1i} ; Z_{ik} is a vector of fixed covariates for each individual *i* that are used to predict level-1 intercepts and slopes, which can include socio-demographic variables among others. In this analysis, mixed-effects regression models did not include exposures (X_{ij}) or covariates (Z_{ij}). They were only used to predict empirical bayes estimators for baseline cognitive performance for each test, with *TIME*

as the only variable in the multi-level model. ζ_{0i} and ζ_{1i} are level-2 disturbances; ε_{ij} is the within-person level-1 disturbance [12].

It is worth noting that the models were fit using the entire HANDLS cohort with complete data on either v1 or v2 on cognitive tests was used to improve reliability of predicted estimates. Finally, empirical Bayes estimators of the intercept π_{0i} were predicted from the simple model with no covariates by adding the fixed effect of the intercept (i.e. γ_{00}) to its predicted individual-level random effect ζ_{0i} thus allowing for imputation of missing data for individuals with only 1 repeat. This baseline cognitive performance score for each test is heretofore labelled as CP.

Methods S3: Principal components analysis of cognitive performance scores

Following this estimation, baseline performance on each cognitive test score were entered into a principal components analysis (PCA) as measured variables [13] in which a number of common factors were extracted based on common variance, component loadings estimated and the residual variance labeled as uniqueness for each LARCC. The principal component analysis model can be summarized as follows:

$$CP_i = \sum_{j=1}^{k} \lambda_{ij} * Domain_j + \varphi_i$$

Where CP_i is the standardized z-score for each predicted baseline cognitive performance test score, λ_{ij} is the component loading for each CP_i and each factor, $Domain_j$ is the standardized z-score for each factor j, and φ_i is the residual error, the squared value of which is the uniqueness. The sum of squared factor loadings for each CP_i is the communality or the common variance that is accounted for by the extracted factors.

An eigenvalue>1 rule was used and the scree plot was observed to determine the adequate number of extracted components that would produce the best model fit, particularly that would explain the greatest amount of variance in the data. The component loadings were then rotated using varimax orthogonal rotation and the factors were interpreted, and cognitive domains labeled accordingly, with cutoff point of 0.30 or more for significant loading. The component scores (*z*-scores) were predicted and used as markers of CP_i for specific cognitive domains. Note that all CPi were entered in the direction of greater score → better performance. Thus, CP_i of BVRT, TRAILS A and B were multiplied by -1, prior to inclusion in the PCA model (See **Figures S3** and **S4** and **Tables S3** and **S4** for main results).

Results S1: Detailed results

The inclusion criteria for the three analytic samples (i.e., **Sample 1**, **Sample 2**, **Sample 3** in **Figure S1**) yielded some differences in their characteristics relative to the initial study cohort (n=3,720). For example, the individuals comprising **Sample 1** were older (mean [standard error]: 48.5 [0.19] vs 47.8 [0.25], $p = 0.0157$) and more likely female (57.6% vs 50.4%, $p < 0.001$) than those excluded from **Sample 1**. Individuals for whom *APOE* genotype information was available (**Sample 2**) were older (mean [standard error]: 48.6 [0.19] vs 47.84 [0.26], $p < 0.001$) and more likely White (44.5% vs 34.9%, $p < 0.001$) than those without *APOE* genotype information. The individuals comprising **Sample 3** were older (mean [standard error]: 48.5 [0.22] vs 47.9 [0.22], $p = 0.0308$), more likely female (57.2% vs 52.6%, $p = 0.006$), less likely below poverty (38.9% vs 43.3%, $p = 0.006$), and more likely White (44.9% vs 37.4%, $p < 0.001$) than those excluded from **Sample 3.**

The proportion of African Americans living below poverty was higher than it was for Whites (<125% federal poverty line: 45% vs. 31%). Moreover, a with lower proportion of African

Americans attained a level of education above HS (34% vs. 36%). On average, African American adults had lower levels of literacy as determined by the WRAT-3 literacy (40.8 vs. 44.8, $p<0.05$). Racial differences were also observed with respect to current use of drugs (21.1% in African Americans vs. 13.1% among Whites), poor/average self-rated health (21.9% in African Americans vs. 26.7% in Whites), very good/excellent self-rated health (32.9% in African Americans vs. 35.8% in Whites), CES-D total score (14.3 in African Americans vs. 15.5 in Whites), hypertension (50.8% in African Americans vs. 40.3% in Whites), dyslipidemia (24.9% in African Americans vs. 28.8% in Whites), and CVD (19.4% in African Americans vs. 15.2% among Whites).

Supplemental references:

1 Folstein, M. F., Folstein, S. E. & McHugh, P. R. "Mini-mental state". A practical method for grading the cognitive state of patients for the clinician. *J Psychiatr Res* **12**, 189-198 (1975).

2 Delis, D. C., Freeland, J., Kramer, J. H. & Kaplan, E. Integrating clinical assessment with cognitive neuroscience: construct validation of the California Verbal Learning Test. *J Consult Clin Psychol* **56**, 123-130 (1988).

3 Benton, A. L. (The Psychological Corportation, New York, 1974).

4 Wechsler, D. WAIS-R manual., (The Psychological Corporation, Cleveland, 1981).

5 Morris, J. C. *et al.* The Consortium to Establish a Registry for Alzheimer's Disease (CERAD). Part I. Clinical and neuropsychological assessment of Alzheimer's disease. *Neurology* **39**, 1159-1165 (1989).

6 Morris, J. C., Mohs, R. C., Rogers, H., Fillenbaum, G. & Heyman, A. Consortium to establish a registry for Alzheimer's disease (CERAD) clinical and neuropsychological assessment of Alzheimer's disease. *Psychopharmacol Bull* **24**, 641-652 (1988).

7 Schretlen, D., Bobholz, J. H. & Brandt, J. Development and psychometric properties of the Brief Test of Attention. *Clinical Neuropsychologist* **10**, 80-89 (1996).

8 Reitan, R. *Trail Making Test: Manual for Administration and Scoring*. (Reitan Neuropsychological Laboratory, 1992).

9 Rouleau, I., Salmon, D. P., Butters, N., Kennedy, C. & McGuire, K. QUANTITATIVE AND QUALITATIVE ANALYSES OF CLOCK DRAWINGS IN ALZHEIMERS AND HUNTINGTONS-DISEASE. *Brain and Cognition* **18**, 70-87 (1992).

10 Wilkinson, G. S. *Wide Range Achievement Test–Revision 3*. (Jastak Association, 1993).

11 Nguyen, H. T., Kitner-Triolo, M., Evans, M. K. & Zonderman, A. B. Factorial invariance of the CES-D in low socioeconomic status African Americans compared with a nationally representative sample. *Psychiatry Res* **126**, 177-187, doi:10.1016/j.psychres.2004.02.004 (2004).

12 Blackwell, E., de Leon, C. F. & Miller, G. E. Applying mixed regression models to the analysis of repeated-measures data in psychosomatic medicine. *Psychosom Med* **68**, 870-878, doi:10.1097/01.psy.0000239144.91689.ca (2006).

13 Sharma, S. *Applied multivariate techniques*. (Wiley, 1996).

Table S1. Study sample characteristics (Cont'd), overall and by race for sub-sample with complete and valid cognitive performance data at either visit and *APOE* genotype data, excluding participants who died during first year, HANDLS 2004-2009 [a]

	**Overall**	**Whites**	**African American**
	(X ± SE), %	(X ± SE), %	(X ± SE), %
	**(N=1,770)**	**(N = 794)**	**(N = 976)**
APOE genotype, %			
e3/e3	51.1 ± 1.2	61.1 ± 1.7	43.0 ± 1.6
e2/e2	0.7 ± 0.2	0.5 ± 0.3	0.9 ± 0.3
e2/e3	14.9 ± 0.8[d***]	12.6 ± 1.2	16.7 ± 1.2
e2/e4	4.0 ± 0.5[d***]	2.4 ± 0.5	5.2 ± 0.7
e3/e4	25.4 ± 1.0[d***]	21.2 ± 1.4	28.9 ± 1.5
e4/e4	3.9 ± 0.5[d***]	2.3 ± 0.5	5.2 ± 0.7
Baseline drug and tobacco use			
Any drug, current user, %	17.5 ± 0.9[d***]	13.1 ± 1.3	21.1 ± 1.3
Tobacco, current user, %	44.8 ± 1.2	43.1 ± 1.8	46.2 ± 1.6
Body mass index, kg/m^2	30.127 ± 0.183	30.288 ± 0.268	29.996 ± 0.251
Self-rated health, %			
Poor/Average,	24.1 ± 1.0[d**]	26.7± 1.6	21.9 ± 1.3
Good	41.7 ± 1.2	37.5 ± 1.7	45.2 ± 1.6
Very good/Excellent	34.2 ± 1.1[d*]	35.8 ± 1.7	32.9 ± 1.5
HEI-2010 total score at v_1	42.5278 ± 0.306	42.184 ± 0.471	42.806 ± 0.392
Total energy intake, kcal/day	2017.636 ± 24.407	2034.608 ± 37.680	2003.828 ± 35.118
CES-D total score	14.877 ± 0.271[d*]	15.509 ± 0.417	14.364 ± 0.356

Hypertension[b], %	45.9 ± 1.2[d***]	40.3 ± 1.8	50.8 ± 1.6
Diabetes[b], %			
No	64.3 ± 1.1	63.3 ± 1.7	65.1 ± 1.5
Pre-diabetic	18.6 ± 0.9	20.8 ± 1.5	16.8 ± 1.2
Diabetic	17.1 ± 0.9	15.9 ± 1.3	18.1 ± 1.2
Dyslipidemia[b], %	28.8± 1.1[d***]	33.6 ± 1.8	24.9 ± 1.5
Cardiovascular disease[b], %	17.6 ± 0.9[*]	15.1 ± 1.3	19.7 ± 1.3
Co-morbidity index[b]	1.456 ± 0.034	1.423 ± 0.051	1.482 ± 0.046
Cognitive performance at v_1, unadjusted[c]			
MMSE	27.772 ± 0.051[4***]	28.163 ± 0.075	27.454 ± 0.069
CVLT-List A	24.635 ± 0.167[d***]	26.044 ± 0.263	23.541 ± 0.207
CVLT-DFR	7.321 ± 0.079[d***]	8.131 ± 0.121	6.690 ± 0.099
BVRT	6.339 ± 0.118[*]	6.062 ± 0.167	6.566 ± 0.166
BTA	6.692 ± 0.055[d***]	7.199 ± 0.079	6.287 ± 0.072
AF	18.866 ± 0.127[d***]	19.776 ± 0.198	18.125 ± 0.160
DS-F	7.317 ± 0.052[d***]	7.633 ± 0.081	7.058 ± 0.067
DS-B	5.692 ± 0.052[d***]	6.234 ± 0.083	5.246 ± 0.063
CDT	8.799 ± 0.029[d***]	8.972 ± 0.041	8.656 ± 0.039
TRAILS A	36.392 ± 0.915[d***]	31.690 ± 0.831	40.246 ± 1.508
TRAILS B	143.723 ± 3.702[d***]	111.639 ± 4.573	170.019 ± 5.455

Abbreviations: AF=Animal Fluency; *APOE*=Apolipoprotein E genotype; BMI=Body Mass Index; BTA=Brief Test of Attention; BVRT=Benton Visual Retention Test; CDT=Clock Drawing Test; CES-D=Center for Epidemiologic Studies-Depression; CVLT-DFR=California Verbal Learning Test-Delayed Free Recall; CVLT-List A=California Verbal Learning Test-List A; DS-B=Digits Span-Backward; DS-F=Digits Span-Forward; HANDLS = Healthy Aging in Neighborhood of Diversity across the Lifespan; HEI-2010=Healthy Eating Index, 2010 version; HS = High school; MMSE=Mini-Mental State Examination; PCA=Principal Components Analysis; SD=Standard Deviation; TRAILS A=Trailmaking Test, Part A; TRAILS B=Trailmaking Test, part B; WRAT-3 = Wide Range Achievement Test, 3rd revision; X = mean.

[a]Values are means (X) ±SE for continuous variables and % for categorical variables. The sample selected has complete data on MMSE and 10 other cognitive test scores at visits 1 and/or 2 and complete data on *APOE* genotypes. Other covariates were multiple imputed (k=5 imputations), using chained equations. All cognitive test scores are in the direction of higher score → better performance with the exception of BVRT (# of errors) and TRAILS A and B (# of sec. to complete). All measures presented in this Table are v_1 measures (2004-2009).

[b]The co-morbidity index was calculated as the sum of hypertension, diabetes and dyslipidemia (or statin use), and self-reported history of cardiovascular disease included atrial fibrillation, angina, coronary artery disease, congestive heart failure, or myocardial infarction, ranging from 0 to 5.

[c]Crude baseline cognitive test score.

[d]$p<0.05$ upon further adjustment for age, sex, and poverty status in multiple linear, logistic and multinomial logit models with race entered as the main predictor.

*$p < 0.05$** $p < 0.01$; *** $p < 0.001$, *t*-test for null hypothesis of no between-race differences.

Table S2. Varimax rotated two-factor solution of predicted baseline cognitive performance using 10 cognitive test scores CP_i as measured variables, N=2,289

		PCA1	PCA2	PCA3
CVLT-LIST A		**0.6341**	-0.0018	-0.0410
CVLT-DFR		**0.6551**	-0.0614	-0.0273
BVRT		0.1438	0.0658	**0.3773**
BTA		0.0140	**0.3298**	0.2381
AF		0.3561	0.0928	0.0318
DS-F		-0.0678	**0.6832**	-0.0903
DS-B		0.0314	**0.6135**	-0.0177
CDT		0.0159	0.0629	**0.3698**
TRAILS A		-0.1008	-0.1424	**0.6750**
TRAILS B		0.0711	0.0842	**0.4435**
Eigenvalue		2.23	2.00	1.79
% variance explained		22%	20%	18%

Abbreviations: AF=Animal Fluency; BTA=Brief Test of Attention; BVRT=Benton Visual Retention Test; CDT=Clock Drawing Test; CVLT-DFR=California Verbal Learning Test-Delayed Free Recall; CVLT-List A=California Verbal Learning Test-List A; DS-B=Digits Span-Backward; DS-F=Digits Span-Forward; PCA=Principal Components Analysis; TRAILS A=Trailmaking Test, Part A; TRAILS B=Trailmaking Test, part B.

Table S3. Correlation matrix of PCA1, PCA2 and PCA3

		PCA1	PCA2	PCA3
PCA1		1		
PCA2		0.45	1	
PCA3		0.46	0.46	1

Abbreviations: PCA=Principal components analysis.

Table S4. Interactive associations of cognitive performance and *APOE4* dosage with all-cause and CVD mortality in the overall sample, Cox proportional hazards models: HANDLS 2004-2018 [a]

	All-cause mortality		CVD mortality	
	(N=1,770)		(N =1,770)	
	n=260 deaths		n=76 deaths	
	**Log_e(HR)**	**(SE)**	**Log_e(HR)**	**(SE)**
**Models 1B: MMSE interaction with *APOE4***				
MMSE	-0.05	0.05	-0.01	0.09
APOE4	-0.64	1.69	-3.36	3.29
MMSE× *APOE4*	0.02	0.06	0.11	0.12
**Models 2B: verbal memory/fluency domains (**PCA1**) interaction with *APOE4***				
PCA1	0.02	0.06	-0.07	0.11
APOE4	-0.04	0.12	-0.09	0.23
PCA1× *APOE4*	0.24	0.09	0.21	0.16
**Models 3B: Attention and working memory domains (**PCA2**) interaction with *APOE4***				
PCA2	0.05	0.07	-0.10	0.13
APOE4	-0.05	0.11	-0.15	0.22
PCA2× *APOE4*	0.08	0.08	0.13	0.16

**Models 4B: Executive function and visuo-spatial domains (**PCA3) **interaction with *APOE4***				
PCA3	-0.06	0.06	-0.23**	0.08
APOE4	-0.06	0.12	-0.11	0.23
PCA3× *APOE4*	0.01	0.07	0.15	0.11

Abbreviations: APOE=Apolipoprotein E gene; BMI=Body Mass Index; CES-D=Center of Epidemiological Studies-Depression; CVD=Cardiovascular Disease; HANDLS = Healthy Aging in Neighborhood of Diversity across the Lifespan; HEI-2010=Healthy Eating Index, 2010; HS = High school; MMSE=Mini-Mental State Examination; PCA=Principal Components Analysis; WRAT-3 = Wide Range Achievement Test, 3rd revision.

[a] Models included each of 4 cognitive performance variables separately as the main predictor for all-cause or CVD mortality and interacted this main predictor with ApoE4 dosage. The models were carried out in the overall population only. All models adjusted only for age, sex, race, poverty status, education and the WRAT-3 score using imputed data, in addition to other lifestyle and health-related factors, namely current drug use, current tobacco use, body mass index, self-rated health, co-morbidity index, HEI-2010, total energy intake, CES-D total score, and the inverse mills ratio.

*$p < 0.05$** $p < 0.01$; *** $p < 0.001$, *t*-test for null hypothesis of $Log_e(HR)=0$.

Table S5. Association between HDL and Total Cholesterol allostatic load components at v_1 and *APOE2* or *APOE4* dosages: Multiple logistic regression models, HANDLS 2004-2009 [a]

	**AL component Elevated total Cholesterol (≥240 mg/dL)**	**AL component Reduced HDL Cholesterol (<40 mg/dL)**
	X ± SE	X ± SE
	**(N=1,714)**	**(N =1,714)**
	**Log_e(OR)±SE**	**Log_e(OR)±SE**
MODEL 1		
APOE2	-0.42*** ± 0.22	-0.39* ± 0.17
MODEL 2		
APOE4	+0.49*** ± 0.14	+0.16* ± 0.12

Abbreviations: AL=Allostatic Load; APOE=Apolipoprotein E genotype; HANDLS = Healthy Aging in Neighborhood of Diversity across the Lifespan; HDL= High-density lipoprotein; SE=Standard Error; X = Coefficient Estimate.

[a] All models adjusted for age, sex, race, poverty status, education and the WRAT-3 score using imputed data, in addition to other lifestyle and health-related factors, namely current drug use, current tobacco use, body mass index, self-rated health, co-morbidity index, HEI-2010, total energy intake, CES-D total score, and the inverse mills ratio.

*p < 0.05** p < 0.01; *** p < 0.001, t-test for null hypothesis of no between-race differences.

Table S6. Interactive associations of cognitive performance and *APOE2* with all-cause and CVD mortality in the overall sample with adjustment for HDL and Total Cholesterol AL, Cox proportional hazards models: HANDLS 2004-2018 [a]

	**All-cause mortality**		**CVD mortality**	
	**(N=1,770)**		**(N =1,770)**	
	**n=260 deaths**		**n=76 deaths**	
	**Log_e(HR)**	**(SE)**	**Log_e(HR)**	**(SE)**
**Models 1A: MMSE interaction with *APOE2***				
MMSE	-0.02	0.05	0.08	0.10
APOE2	3.40	2.14	3.35	4.47
MMSE× *APOE2*	-0.13	0.08	-0.14	0.17
HDL AL	0.29	0.16	0.15	0.29
Total Cholesterol AL	-0.17	0.22	0.06	0.36
**Models 2A: verbal memory/fluency domains (**PCA1): **interaction with *APOE2***				
PCA1	0.01	0.06	-0.03	0.11
APOE2	-0.06	0.16	-0.47	0.36
PCA1× *APOE2*	0.04	0.11	0.06	0.26
HDL AL	0.29	0.16	0.17	0.29
Total Cholesterol AL	-0.14	0.22	0.14	0.35
**Models 3A: Attention and working memory domains (**PCA2): **interaction with *APOE2***				
PCA2	0.13*	0.06	0.04	0.11
APOE2	-0.19	0.17	-0.99*	0.46
PCA2× *APOE2*	-0.33**	0.13	-0.78*	0.32
HDL AL	0.28	0.16	0.12	0.29
Total Cholesterol AL	-0.15	0.22	0.10	0.35
**Models 4A: Executive function and visuo-spatial domains (**PCA3): **interaction with *APOE2***				
PCA3	-0.04	0.05	-0.12	0.07

APOE2	-0.12	0.17	-0.93*	0.46
PCA3× *APOE2*	-0.23	0.13	-0.69*	0.27
HDL AL	0.31	0.16	0.18	0.30
Total Cholesterol AL	-0.16	0.22	0.03	0.35

Abbreviations: AL=Allostatic Load; APOE=Apolipoprotein E gene; BMI=Body Mass Index; CES-D=Center of Epidemiological Studies-Depression; CVD=Cardiovascular Disease; HANDLS = Healthy Aging in Neighborhood of Diversity across the Lifespan; HDL= High-density lipoprotein; HEI-2010=Healthy Eating Index, 2010; HS = High school; MMSE=Mini-Mental State Examination; PCA=Principal Components Analysis; WRAT-3 = Wide Range Achievement Test, 3rd revision.

[a] Models included each of 4 cognitive performance variables separately as the main predictor for all-cause or CVD mortality and interacted this main predictor with ApoE2 dosage. The models were carried out in the overall population only. All models adjusted only for age, sex, race, poverty status, education and the WRAT-3 score using imputed data, in addition to other lifestyle and health-related factors, namely current drug use, current tobacco use, body mass index, self-rated health, co-morbidity index, HEI-2010, total energy intake, CES-D total score, and the inverse mills ratio.

*$p < 0.05$**; $p < 0.01$; *** $p < 0.001$, t-test for null hypothesis of $\text{Log}_e(\text{HR})=0$.

Table S7. Interactive associations of cognitive performance and *APOE4* dosage with all-cause and CVD mortality in the overall sample with adjustment for HDL and Total Cholesterol AL, Cox proportional hazards models: HANDLS 2004-2018 [a]

	All-cause mortality		CVD mortality	
	(N=1,770)		(N =1,770)	
	n=260 deaths		n=76 deaths	
	Log_e(HR)	(SE)	Log_e(HR)	(SE)
**Models 1A: MMSE interaction with *APOE4***				
MMSE	-0.06	0.05	0.02	0.10
APOE4	-0.59	1.70	-3.80	3.40
MMSE× *APOE4*	0.02	0.06	0.13	0.12
HDL AL	0.30	0.16	0.21	0.39
Total Cholesterol AL	0.01	0.01	0.01	0.02
**Models 2A: verbal memory/fluency domains (**PCA1): **interaction with *APOE4***				
PCA1	0.00	0.62	-0.10	0.12
APOE4	-0.01	0.12	-0.08	0.23
PCA1× *APOE4*	0.05	0.90	0.22	0.17
HDL AL	0.30	0.16	0.21	0.30
Total Cholesterol AL	-0.12	0.22	0.21	0.35
**Models 3A: Attention and working memory domains (**PCA2): **interaction with *APOE4***				
PCA2	0.04	0.07	-0.09	0.13
APOE4	-0.03	0.12	-0.15	0.23
PCA2× *APOE4*	0.07	0.08	0.11	0.16
HDL AL	0.30	0.16	0.22	0.30
Total Cholesterol AL	-0.12	0.22	0.20	0.35
**Models 4A: Executive function and visuo-spatial domains (**PCA3): **interaction with *APOE4***				
PCA3	-0.07	0.06	-0.30**	0.01

APOE4	-0.04	0.12	-0.09	0.24
PCA3× *APOE4*	0.02	0.07	0.21	0.12
HDL AL	0.30	0.16	0.21	0.30
Total Cholesterol AL	-0.14	0.22	0.13	0.35

Abbreviations: AL=Allostatic Load; APOE=Apolipoprotein E gene; BMI=Body Mass Index; CES-D=Center of Epidemiological Studies-Depression; CVD=Cardiovascular Disease; HANDLS = Healthy Aging in Neighborhood of Diversity across the Lifespan; HDL= High-density lipoprotein; HEI-2010=Healthy Eating Index, 2010; HS = High school; MMSE=Mini-Mental State Examination; PCA=Principal Components Analysis; WRAT-3 = Wide Range Achievement Test, 3rd revision.

[a] Models included each of 4 cognitive performance variables separately as the main predictor for all-cause or CVD mortality and interacted this main predictor with ApoE2 dosage. The models were carried out in the overall population only. All models adjusted only for age, sex, race, poverty status, education and the WRAT-3 score using imputed data, in addition to other lifestyle and health-related factors, namely current drug use, current tobacco use, body mass index, self-rated health, co-morbidity index, HEI-2010, total energy intake, CES-D total score, and the inverse mills ratio.

*$p < 0.05$; ** $p < 0.01$; *** $p < 0.001$, *t*-test for null hypothesis of $\text{Log}_e(\text{HR})=0$.

FIGURE S1. Participant Flowchart

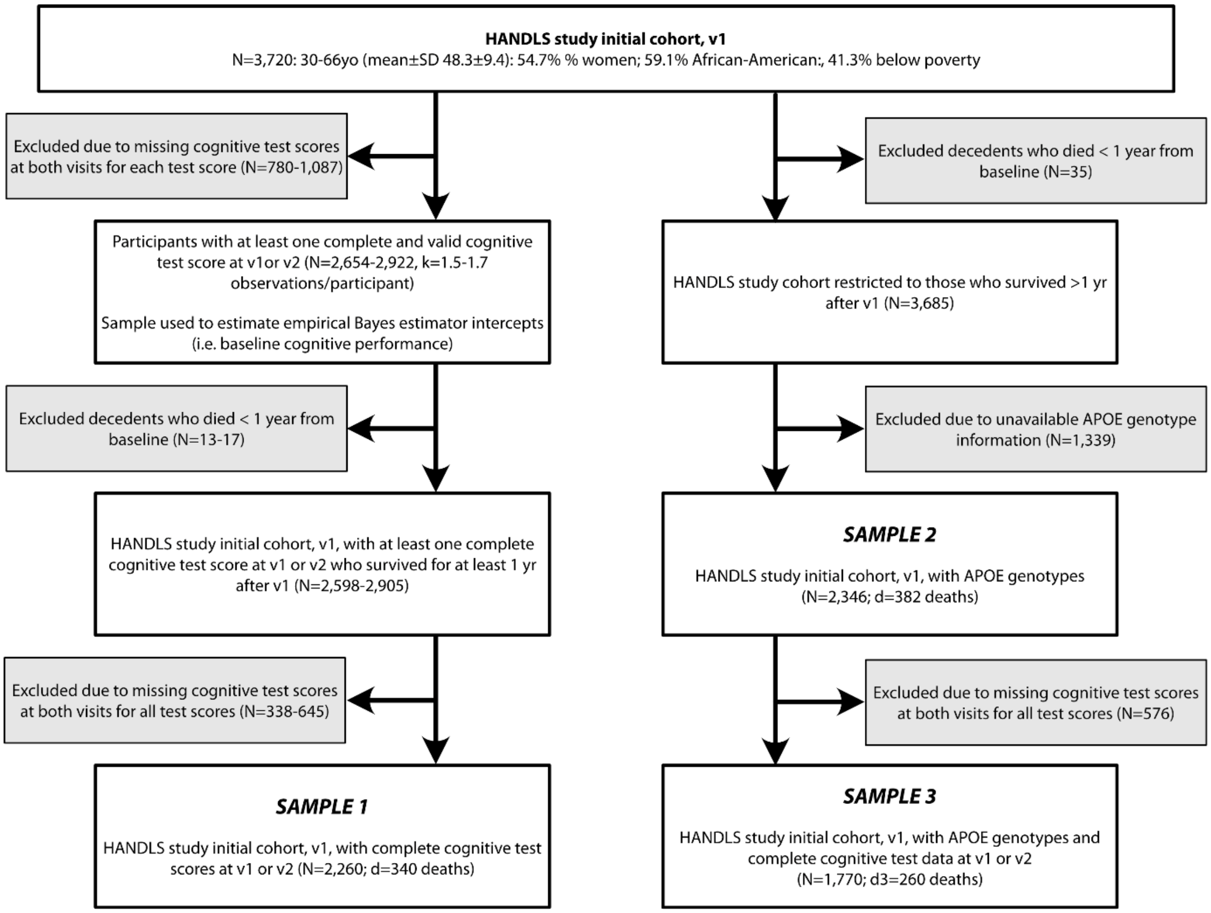

Abbreviations: *APOE*=Apolipoprotein E; HANDLS=Healthy Aging in Neighborhoods of Diversity Across the Life Span.

FIGURE S2. CVD Mortality smoothed hazard rates by *APOE4* dosage: HANDLS 2004-2018

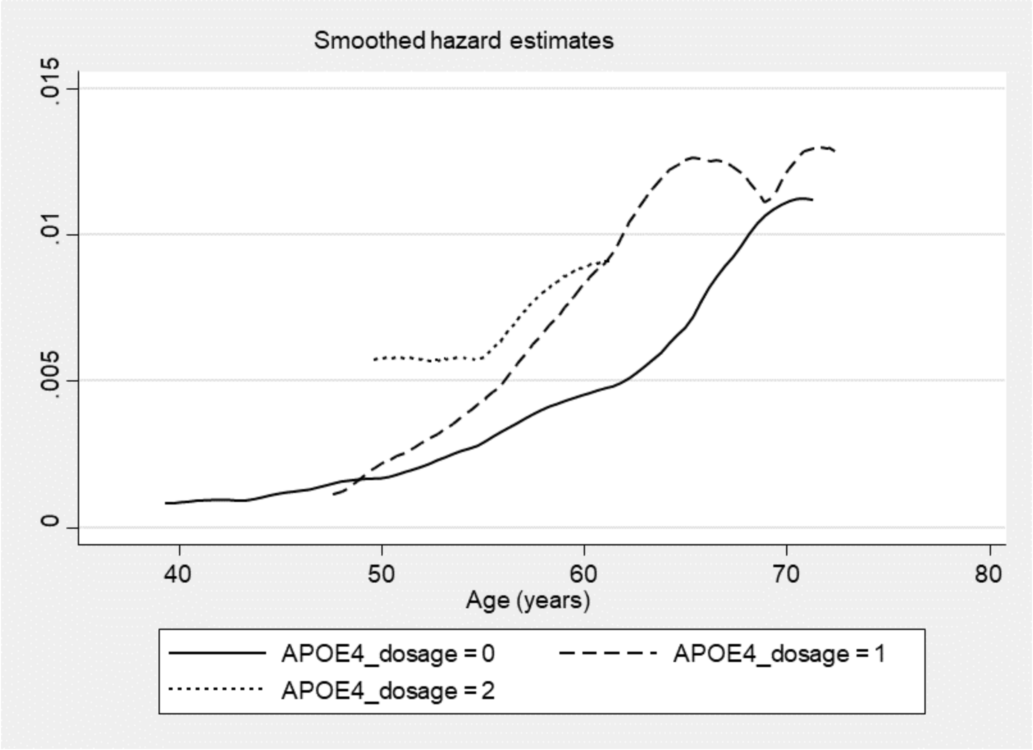

Abbreviations: *APOE4*=Apolipoprotein E ε4 dosage; HANDLS=Healthy Aging in Neighborhoods of Diversity

Across the Life Span

Notes: *APOE4* dosage (coded as 0=no e4 alleles, 1=1 e4 allele, 2=2 e4 alleles) is used as the main predictor for CVD mortality to estimate smoothed hazard rates.
Test for trend for surivivor function: chi2(1) = 4.63 Pr>chi2 =   0.0313

FIGURE S3. Scree plot for PCA of 10 empirical bayes estimators for cognitive performance at baseline; HANDLS 2004-2013

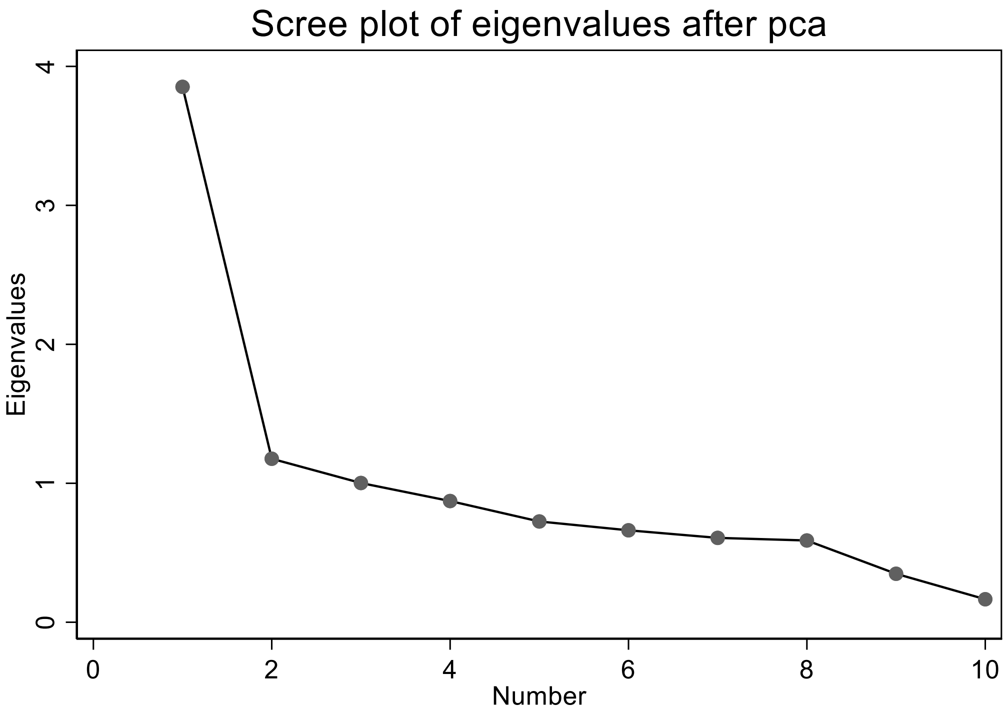

Abbreviations: HANDLS=Healthy Aging in Neighborhoods of Diversity Across the Life Span; PCA=Principal Components Analysis

Figure S4. Scatter plot of PCA1, PCA2 and PCA3; HANDLS 2004-2013

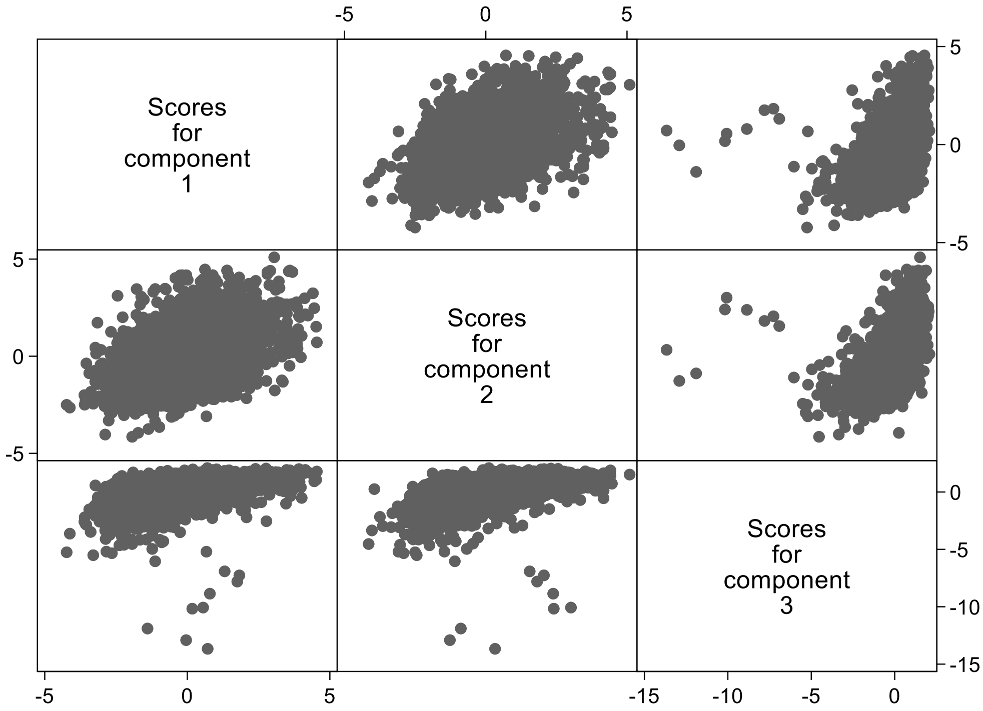

Abbreviations: HANDLS=Healthy Aging in Neighborhoods of Diversity Across the Life Span; PCA=Principal Components Analysis; Scores for component 1=PCA1; Scores for component 2=PCA2; Scores for component 3=PCA3
